# Comparison of Fungal Culture, DermaGenius^®^ Multiplex Real-Time PCR, and the EUROArray Dermatomycosis Assay for the Diagnosis and Species Identification of Dermatophytes

**DOI:** 10.3390/jof12020088

**Published:** 2026-01-28

**Authors:** Felix Lötsch, Theresa Hillinger, Brigitte Selitsch, Kathrin Spettel, Birgit Willinger

**Affiliations:** 1 Division of Clinical Microbiology, Department of Laboratory Medicine, Medical University of Vienna, 1090 Vienna, Austria; 2 Comprehensive Center for Infection Medicine, Medical University of Vienna, Währinger Gürtel 18-20, 1090 Vienna, Austria

**Keywords:** dermatophytes, multiplex PCR, fungal culture, diagnostics

## Abstract

Dermatophytes are fungi that infect the human skin and its appendages. With new pathogenic species emerging and resistance to first-line drugs rising, microbiologic diagnosis and species identification are becoming even more important. In this study, the DermaGenius^®^ 2.0/3.0 Complete multiplex real-time PCR and the EUROArray Dermatomycosis kits were compared to fungal culture and with each other; 78 reference strains and 124 clinical samples were analyzed. Both the DermaGenius^®^ kit (97%; 95%CI 89–100%) and the EUROArray assay (91%; 95% CI: 82–96%) were sensitive when analyzing on-panel reference strains. In clinical samples, the DermaGenius^®^ assay provided a positive result in 63 out of 124 (51%) samples and the EUROArray assay in 74 out of 124 (60%) samples. Both kits supported the diagnosis and species identification of culture-negative samples, and samples with growth of unconventional species. However, there was suspicion of false-positive results with *F. solani* in the EUROArray kit both in clinical and reference strains. The most common conventional dermatophytes in this study combining all methods were *T. rubrum*/*soudanense* (n = 40) and *T. interdigitale*/*mentagrophytes* (n = 11). In summary, both PCR kits were sensitive for the diagnosis and species identification of dermatophytoses. Combining culture and a PCR-based method can increase the diagnostic yield and compensate for the weakness of the other methods. The optimal PCR-based kit, and especially the optimal panel size, depends on the local epidemiology of dermatophytes.

## 1. Introduction

Dermatophytes are fungi that can infect human skin and its appendages, such as hair or nails. Such infections, known as dermatophytoses, are highly prevalent across the globe and affect people of all ages, including children [1]. Although most infections are relatively harmless, they can cause disfigurement, increase susceptibility to bacterial skin infections, and significantly impact patients’ quality of life [2]. This underscores the need for a rapid diagnosis and effective treatment. Various species from different genera, notably *Trichophyton* and *Microsporum*, among others, are responsible for dermatophytoses [3]. Due to the broad spectrum of involved genera and species, identifying the underlying pathogen is therefore not trivial, particularly when relying on conventional fungal culture. Additionally, even some yeasts and certain molds may lead to similar conditions, and emerging pathogenic dermatophytes have been recognized in recent years, such as *Nannizia nana* for example [4,5]. In clinical practice, the diagnosis of dermatophytoses is usually based on clinical grounds, and treatment is often prescribed without any microbiological testing. However, besides an ever-increasing complexity in pathogenic genera and species, growing resistance to first-line antifungals is emerging as a problem. Certain species are notorious, such as terbinafine-resistant *Trichophyton indotineae* (Trichophyton mentagrophytes ITS genotype VIII), which has caused an epidemic of difficult-to-treat infections in India [6], but has also been found in Europe and other parts of the world [7,8]. Therefore, rapid species identification and phenotypic resistance testing or molecular detection of resistance markers are rapidly gaining importance. Traditionally, microscopy and fungal culture have long been considered the method of choice. Unfortunately, fungal culture and antifungal susceptibility testing are laborious, time-consuming, and dependent on the skills of the technician [9]. As a result, molecular tests, such as multiplex PCR panels, that enable rapid species identification, have become important tools for clinical routine settings [10,11,12]. However, the diagnostic yield of many PCR-based kits is limited by the range of the panel, with emerging or rare species going undetected.

In this study, we compared fungal culture with two commercially available test kits (DermaGenius^®^2.0/3.0 Complete Multiplex real-time PCR and the EUROArray Dermatomycosis kit) for species identification. We then assessed the possible benefit of combining these methods. The EUROArray Dermatomycosis kit is a microarray test system based on PCR. Its panel allows the identification of 23 different species of dermatophytes plus a pan-dermatophyte target, three yeasts, and three mold species, allowing in total the detection of 50 dermatophyte species. The DermaGenius^®^ 2.0 Complete Multiplex real-time PCR kit is based on melting curve analysis and allows the identification and differentiation of ten different species complexes of dermatophytes plus *Candida albicans* (panels see Table 1). In the 3.0 version of the kit, additional targets for *N. gypsea*, *C. parapsilosis*, *S. brevicaulis*, and a pan-dermatophyte target were added. For this study, we tested samples from cultured reference strains and patient material and assessed the diagnostic performance of the kits and combinations thereof.

## 2. Materials and Methods

For this study, quality control samples and left-over samples previously collected and stored after routine diagnostic methods were used. Ethical clearance was granted by the Ethics Committee of the Medical University of Vienna (EK 1047/2020).

### 2.1. Samples

Reference strains consisted of a collection of 78 well-characterized isolates that were previously used in round robin tests provided by INSTAND and NEQAS for quality control purposes (see also Table 2). DNA was extracted between 2020 and 2021 and stored at −80 °C. Clinical samples were 124 left-over samples after standardized routine diagnostics had been performed. They were collected between 2020 and 2023 at the University Hospital Vienna (AKH Wien) and stored at −20 °C until further use if PCR could not be performed immediately. Samples were stored in non-frost-free freezers under a stringent quality control system with continuous monitoring. No freezer failures, defrost cycles, or temperature deviations occurred over the years of storage. As nomenclature and taxonomy is rapidly developing and to facilitate comparability between assays, we grouped closely related and developing species into groups. *Trichophyton rubrum* and *T. soudanense* were grouped as the *T. rubrum*/*soudanense* group, *Trichophyton interdigitale* and *T. mentagrophytes* were grouped as the *T. interdigitale*/*mentagrophytes* group, and *T. erinacei* and *T. benhamiae* were grouped as the *T. benhamiae*/*erinacei* group. *T. indotinae*, an emerging species [8], was neither part of the collection of reference strains nor identified in a clinical sample. When referring to culture-based results, we designated *F. solani* and *F. oxysporum* as species complexes. When referring to molecular results, we applied the terminology used by the manufacturer of the product.

### 2.2. Routine Testing

Routine testing was performed according to local standard operating procedures. In summary, material was inoculated onto Sabouraud Dextrose agar supplemented by chloramphenicol and gentamicin (SAB, BD Diagnostics, Heidelberg, Germany) and Mycosel agar (BD Diagnostics, Heidelberg, Germany) and then incubated at 28–30 °C for up to 4 weeks. Species identification was performed by micro- and macroscopic inspection, supplemented by matrix-assisted laser desorption/ionization time-of-flight mass spectrometry (MALDI-TOF MS, Bruker Daltonics, Bremen, Germany) and/or by ITS (internal transcribed spacer) PCR followed by Sanger gene sequencing. The EUROArray Dermatomycosis assay and DermaGenius^®^ were performed in parallel.

### 2.3. DermaGenius^®^ Complete Multiplex Real-Time PCR 2.0 and 3.0

The DermaGenius^®^2.0 Complete Multiplex RT-PCR (PathoNostics B.V., Maastricht, The Netherlands) is a commercial test kit that allows the detection and differentiation of 10 different species. An updated version of the kit (version 3.0) allows the additional detection and differentiation of *Scopulariopsis brevicaulis*, *Candida parapsilosis*, and *Nannizia gypsea* and includes a pan-dermatophyte probe. The kit is a real-time PCR kit including fluorescent probes and species identification, based on melting curve analysis. For most species, the nuclear ribosomal internal transcribed spacer (ITS) region is used, with the exception of *S. brevicaulis* where TEF1α is targeted and *C. albicans* and *C. parapsilosis* where non-coding sequences are used. For DNA extraction, the PathoNostics Extraction kit (PathoNostics B.V., Maastricht, The Netherlands) was used. All procedures were performed according to the manufacturers’ instructions. All reference strains and most clinical samples were tested using version 2.0 of the kit.

### 2.4. EUROArray Dermatomycosis

The EUROArray Dermatomycosis (EUROIMMUN Medizinische Labordiagnostika AG, Lübeck, Germany) is based on multiplex PCR followed by the detection of the resulting fluorescently labeled PCR products through hybridization with immobilized DNA probes in a microarray system. The kit can detect 50 dermatophytes and allows the differentiation of 23 different species. For DNA extraction, the QIAamp^®^ DNA Mini Kit (Hilden, Germany) provided by EUROIMMUN was used. The EUROArray Dermatomycosis kit is licensed for DNA preparations from skin, hair, nail, fungal culture, or formalin-fixed paraffin-embedded material. The kit was used according to the manufacturer’s instructions. To ensure comparability with the DermaGenius^®^ kit, identification of any species within the group was counted as correct (e.g., identification of *T. interdigitale* in a reference strain of *T. mentagrophytes* was considered as correct identification). Both PCR-based tests were performed in parallel.

### 2.5. Statistical Analysis

We calculated sensitivities of both test kits using reference strains both for the overall sample collection and limited to samples that were part of the respective panel. Due to the lack of a clear gold standard, we did not calculate the sensitivity and specificity when analyzing clinical samples but calculated percent agreement between the methods. Statistical analysis was performed using R [13], RStudio [14] and the following packages: epiR [15], ggplot2 [16], dplyr [17], waffle [18], readxl [19], and RColorBrewer [20].

## 3. Results

### 3.1. Reference Strains

A total of 78 reference strains were tested using both the DermaGenius^®^ 2.0 kit and the EUROArray kit. Among these strains, the most common species was *T. interdigitale*/*mentagrophytes* with 19 unique samples (see Table 2).

In total, 61 out of 78 (78%) tested reference strains were species that are part of the DermaGenius^®^ 2.0 panel. In 59 out of 61 (97%; 95%CI 89–100%) on-panel isolates, the DermaGenius^®^ 2.0 kit correctly identified a single fungus. In one isolate of *T. rubrum*/*soudanense*, the kit identified both *T. rubrum*/*soudanense* and *M. audouinii*. Thus, in 60/61 (98%; 95% 91–100%) samples, one correct species was identified. One isolate of T. *interdigitale*/*mentagrophytes* was a false negative in the DermaGenius^®^ 2.0 kit. Reference strains with *C. parapsilosis*, *N. gypsea*, and *S. brevicaulis* were retested using an expanded version of the kit (DermaGenius 3.0), and all samples were positive and correctly identified.

The same set of samples was also tested using the EUROArray assay, which includes all tested species in its panel. In 71 out of 78 (91%; 95% CI: 82–96%) samples, the single correct species was identified, and in 75 out of 78 (96%; 95% CI: 89–99%), one of the identified species was correct. *F. solani* (n = 3) and *F. oxysporum* (n = 1) were the additional fungi incorrectly identified by the EUROArray assay. Interestingly, all three reference strains of *F. oxysporum* species complex were false negatives in the EUROArray kit.

### 3.2. Clinical Samples

In total, there were 124 clinical samples available for analysis. All samples were submitted for microbiologic testing due to clinical suspicion of a dermatophytosis; 59 samples originated from nails, 54 from skin, 9 from hair/the head, 1 from nail plus skin, and 1 was of unknown origin. Six samples (4.8%) could not be analyzed by culture because there was insufficient or no material available. In 65 out of 118 cultured samples (55%), growth could be observed, and in 53 samples (45%), culture remained negative. Discrepancies compared to Table 3 are explained by the fact that samples with detection of more than one species could be counted more than once there.

**Figure 1:**
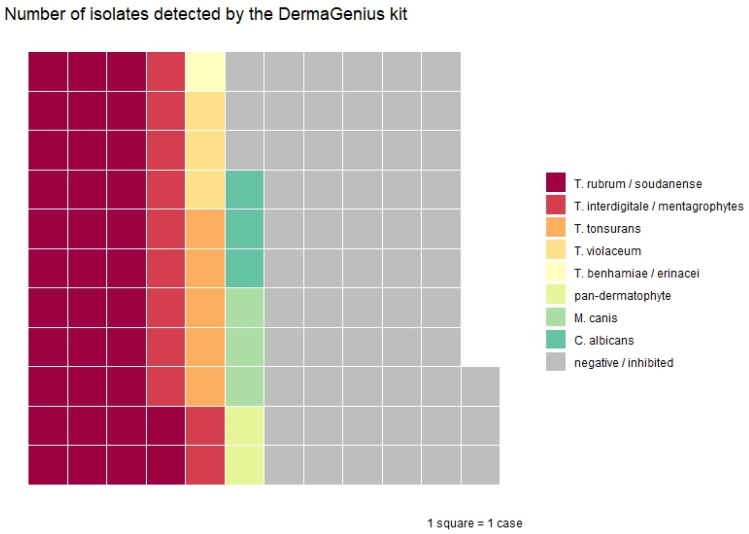
Waffle plot of 63 fungi detected by the DermaGenius kit in clinical samples.

In total, 94 samples were subsequently analyzed by the DermaGenius^®^ 2.0 kit and 30 by the 3.0 version. The DermaGenius^®^ kits provided a positive result for 63 out of 124 samples (51%), 2 of which were positive in the pan-dermatophyte channel only (see Table 3 and Figure 1). One sample was inhibited, and sixty were negative. Interestingly, the DermaGenius^®^ kits were positive in 22 out of 53 samples (42%) that were completely negative in conventional culture: *T. rubrum*/*soudanense* (n = 13), *T. interdigitale*/*mentagrophytes* (n = 7), the pan-dermatophyte target (n = 1), and *C. albicans* (n = 1). The percent agreement between culture and DermaGenius^®^ was 57/118 (48%; 95%CI 39–58%). There was low percent agreement both in culture-negative (58%; 95% CI 44–72%) and culture-positive samples (40%; 95% CI 28–53%). Additionally, in 7 out of 17 samples where culture resulted in unconventional species (*Verticillium* sp., *Epicoccum* sp., *Aureobasidium* sp., *Paecilomyces* sp., *Aspergillus* sp., *Rhodotorula* sp., *Phoma* sp., and/or *Dematiaceae* sp.), the DermaGenius^®^ kit detected a classical dermatophyte: *T. rubrum*/*soudanense* (n = 5), *T. interdigitale*/*mentagrophytes* (n = 1), and the pan-dermatophyte target (n = 1). In four samples with insufficient material for culture, *T. rubrum*/*soudanense* (n = 2), *T. interdigitale*/*mentagrophytes* (n = 1), and *T. tonsurans* (n = 1) were detected. Combining culture and the DermaGenius^®^ assay, 99 fungi were detected in 91 samples (73%).

**Figure 2:**
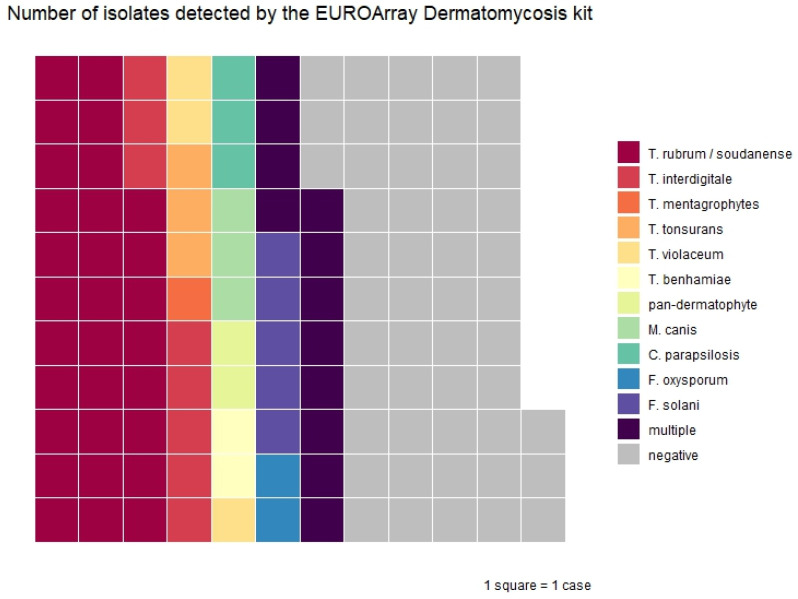
Waffle plot of 74 positive samples by the EUROArray Dermatomycosis kit in clinical samples. Numbers diverge from Table 3, because isolates with multiple positive targets are summarized as “multiple” in this plot.

All 124 samples were also analyzed using the EUROArray kit. The kit was positive in 74/124 samples (60%) (see Figure 2). The EUROArray kit also detected a pathogen in 27 samples that were negative in conventional culture: *T. rubrum*/*soudanense* (n = 10), *T. interdigitale*/*mentagrophytes* (n = 7), *F. solani* (n = 4), *T. rubrum*/*soudanense + F. solani* (n = 2), *C. albicans + F. solani* (n = 1), *F. oxysporum* (n = 1), *T. rubrum*/*soudanense + C. parapsilosis* (n = 1), and *T. rubrum*/*soudanense + F. solani + F. oxysporum* (n = 1). Again, in 7 out 17 samples where culture resulted in unconventional species (see above), the EUROArray kit detected a conventional dermatophyte. These were the same clinical samples where a classical dermatophyte was also detected by the DermaGenius^®^ kit. In one sample with *T. rubrum*/*soudanense* detected by the EUROArray kit, there was insufficient material for culture, and in three samples with *T. rubrum*/*soudanense*, culture showed growth of typical yeasts (n = 2) and *Aspergillus* sp. (n = 1). The overall percent agreement with conventional culture was 51/118 (43%; 95% CI 34–53%), and that with the DermaGenius^®^ kit it was 73% (95% CI 65–81%). Again, there was a low percent agreement both in culture-negative (49%; 95%CI 35–63%) and culture-positive samples (38%; 95% CI 27–51%). Combining culture and the EUROArray led to the detection and identification of 118 fungi in 94 samples (76%). However, in 12 samples, the EUROArray kit detected *F. solani*, which was confirmed by culture in only one case. Moreover, in five of these cases, *F. solani* was detected as a second species next to a conventional dermatophyte, raising the question of contamination.

For quality assurance, reagent controls including DNA extraction reagents were performed 35 times. In four of these, *F. solani* was detected. None of the negative controls, which we performed with every PCR run, were positive.

Discrepancies between the two molecular panels were due to target non-detection by one panel relative to the other. No discrepant single-species identifications were observed (e.g., kit A detecting *Trichophyton tonsurans* while kit B detected *Microsporum canis*).

Combing all test methods, 122 fungi could be identified in 97 samples (78%; 95%CI 70–85%). The most common conventional species were *T. rubrum*/*soudanense* (n = 40) and T. *interdigitale*/*mentagrophytes* (n = 11).

## 4. Discussion

We assessed two commercially available PCR-based assays for the detection and species identification of dermatophytes and other fungi implicated in hair, skin, and nail infections, using both reference strains and clinical specimens. We assessed the diagnostic performance of each PCR method compared with conventional culture and investigated combined testing approaches.

Both test kits performed well with reference strains, correctly identifying more than 90% of samples when only species included in the respective panels were considered. However, the EUROArray kit failed to identify any of the three *F. oxysporum* reference strains. It remains unclear whether this represents a statistical outlier in our study or a true limitation of the assay, warranting independent verification. Furthermore, the EUROArray kit incorrectly identified *F. solani* in three reference strains. Similarly, there were also twelve clinical samples where the EUROArray detected *F. solani* of which only one was confirmed by culture. Although the presence of true-positive, non-culturable isolates cannot be completely excluded, this appears unlikely. A more plausible explanation is contamination either in the DNA extraction kits or in the assay reagents. *F. solani* was detected in 4/35 (11%) reagent control runs (including the DNA extraction reagents provided by the supplier of the kit) but not in negative controls, suggesting contamination of the DNA extraction kits as the most likely explanation. Previous studies have reported inconsistent findings: in a study from Germany using 34 pre-identified isolates, no cases of *F. solani* were detected [21], whereas an Italian study found *F. solani* to be relatively common [11]. In the evaluation study published in the EUROArray kit’s manual, specificity for *F. solani* was the lowest of all species with 80.4%, underlining a possible weak spot of the assay.

In clinical samples, the EUROArray detected and identified fungal species in more samples than either culture or the DermaGenius^®^ kits, likely due to its broader panel but also because of presumably false-positive cases of *F. solani*. Both PCR assays detected and identified pathogens in culture-negative samples, and each detected pathogens missed by the other assay. Moreover, both PCR kits proved useful when culture yielded organisms unlikely to be true pathogens: in 41% (7/17) of such cases, the PCR assays detected conventional dermatophytes. This highlights the role of combining different methods in optimizing the diagnostic yield. Growth of such unconventional species also explains the lower agreement between culture and PCR kits compared to the agreement between both PCR assays. Based on our findings, a combined diagnostic approach yields the highest overall performance, whereas reliance on a single method is suboptimal; while fungal culture remains superior for the detection of uncommon species not included in PCR-based panels, the clinical relevance of such fungi, particularly molds, often remains uncertain and may reflect contamination rather than true infection. Consequently, omitting culture may be acceptable for routine diagnostics in most laboratories, whereas tertiary care centers and reference laboratories should retain culture-based methods to allow detection and characterization of rare or atypical pathogens.

Diagnostic yield, however, is only one consideration when planning for a diagnostic algorithm for clinical samples with suspected fungal infections. The local dermatophyte epidemiology is highly relevant. In our cohort, *T. rubrum*/*soudanense* and *T. interdigitale*/*mentagrophytes* were the most common pathogens both for skin and nail infections, which are both part of the two panels, including the relatively small panel of the DermaGenius^®^ 2.0 kit. These species have also been identified as leading agents of dermatomycoses in comparable studies: Trave et al. found *T. interdigitale* to be the most frequent agent in Italian patients with onychomycosis [11], whereas Romano et al. identified *T. rubrum* more frequently [22]. In Austria, few other studies on the epidemiology of dermatophytoses are available. In one analysis from Graz, by far the most common species identified in cases of tinea capitis was *M. canis* [23], which is also part of both panels.

The most common species in our study not covered by the DermaGenius^®^ kit was *F. solani* (n = 12). Given that the EUROArray incorrectly identified *F. solani* in reference strains and several clinical samples, the true relevance of this species in our collection remains uncertain. The additional yield due to the larger spectrum of the EUROArray kit was low, with only four additional samples identified compared to the DermaGenius^®^ 2.0 panel (*C. parapsilosis* and *N. gypsea*). Both species were added to the updated 3.0 version of the DermaGenius^®^ kit. Nonetheless, the larger EUROArray panel may provide advantages in settings with different epidemiological patterns. Its ability to differentiate closely related species such as *T. mentagrophytes* and *T. interdigitale* also makes it particularly suitable for research applications.

Among all methods, the DermaGenius^®^ assay was the least labor-intensive and provided fastest results. Despite being labor-intensive and relatively insensitive, fungal culture retains the unique advantage of detecting rare or novel pathogens. In this study, for example, *Paecilomyces* species were detected by fungal culture (n = 5), which cannot be identified by either of the panels. Although contamination cannot be excluded and the relevance of this genus in dermatophytoses is not clear, *Paecilomyces* species have been described as potential pathogens [24,25,26].

Both kits were compared in a previous study using previously identified isolates [21], but to our knowledge, our study is the first comparing the DermaGenius^®^ Complete multiplex real-time PCR and the EUROArray Dermatomycosis kits using both reference strains and clinical samples of different origin (skin, nail, and hair). A limitation of our study is that most clinical specimens were tested using DermaGenius^®^ version 2.0 and only a minority with version 3.0, precluding conclusions about performance differences between the versions. Unlike previous studies, we did not calculate specificities or positive predictive values for the PCR assays, because in our view, classifying PCR-positive but culture-negative specimens in patients with a high pretest probability of dermatophytosis as “false positives” is inappropriate. Another limitation is that microscopy was not performed as part of this study. Microscopy is laborious but may provide useful information for distinction between real infection or contamination, especially when rare or uncommon fungi are identified. Furthermore, at the time of this study, emerging *T. indotineae*, an off-shoot of *T. mentagrophytes*, was exceedingly rare at our center. We were therefore also not able to provide data on the methods’ performances in detecting this species.

In summary, in a clinical routine setting, combining a rapid PCR-based method for rapid identification even with a limited panel with culture-based methods is complementary and mitigates the limitations of each approach. The optimal method, kit, or algorithm for diagnosis and species identification of dermatophytoses depends on the local epidemiology, as well as available resources and expertise. Both the DermaGenius^®^ and EUROArray kits are valuable tools in the diagnosis of dermatophytoses when applied in the appropriate clinical context.

## Figures and Tables

**Table 1:** Panel of the Dermagenius 2.0 and 3.0 complete multiplex real-time PCR and the EUROIMMUNE EUROArray dermatomycoses kit. Species marked with an asterisk * are only part of the 3.0 version of the DermaGenius.

DermaGenius Complete Multiplex Real-Time PCR 2.0 and 3.0 *	EUROArray Dermatomycoses
*Trichophyton interdigitale*/*mentagrophytes*	*Trichophyton interdigitale* *Trichophyton mentagrophytes*
*Trichophyton rubrum*/*soudanense*	*Trichophyton rubrum*
*Trichophyton tonsurans*	*Trichophyton tonsurans*
*Trichophyton schoenleinii*/*quinckeanum*	*Trichophyton schoenleinii* *Trichophyton quinckeanum*
*Trichophyton violaceum*	*Trichophyton violaceum*
*Trichophyton benhamiae*	*Trichophyton benhamiae*
*Trichophyton verrucosum*	*Trichophyton verrucosum*
-	*Trichophyton equinum*
-	*Trichophyton concentricum*
-	*Trichophyton bullosum*
-	*Trichophyton simii*
-	*Trichophyton erinacei*
-	*Trichophyton eriotrephon*
*Epidermophyton floccosum*	*Epidermophyton floccosum*
*Microsporum canis*	*Microsporum canis*
*Microsporum audouinii*	*Microsporum audouinii*
-	*Microsporum ferrugineum*
-	*Nannizzia fulva*
Nannizia gypsea *	*Nannizzia gypsea*
-	*Nannizzia incurvata*
-	*Nannizzia persicolor*
*Candida albicans*	*Candida albicans*
Candida parapsilosis *	*Candida parapsilosis*
-	*Candida guilliermondii*
-	*Fusarium solani*
-	*Fusarium oxysporum*
Scopulariopsis brevicaulis *	*Scopulariopsis brevicaulis*
Pan-dermatophyte target *	Pan-dermatophyte target

**Table 2:** Species of the reference strain collection.

Species	n
*Trichophyton interdigitale*/*mentagrophyte*	19
*Trichophyton rubrum*/*soudanense*	14
*Microsporum canis*	6
*Nannizzia gypsea*	5
*Trichophyton benhamiae*	5
*Trichophyton tonsurans*	5
*Epidermophyton floccosum*	4
*Fusarium oxysporum complex*	3
*Trichophyton erinacei*	3
*Trichophyton violaceum*	1
*Microsporum audouinii*	2
*Microsporum fulvum*	2
*Nannizzia persicolor*	2
*Scopulariopsis brevicaulis*	2
*Candida albicans*	1
*Candida (Meyerozyma) guilliermondii*	1
*Candida parapsilosis*	1
*Fusarium solani complex*	1
*Trichophyton verrucosum*/*eriotrephon*	1

**Table 3:** Species detected by each method in clinical samples. “n.a.” = “not applicable” (i.e., not part of the panel); * other cultures species include *Aureobasidium pullulans*, other *Candida* spp. *Rhodotorula* spp., other yeasts, *Aspergillus* spp., *Fusarium* spp., other molds, *Verticillium* spp., *Phoma* spp., *Paecilomyces* spp., *Dematiaceae* spp., and *Epicoccum* spp. The total of each test may be higher than the total number of samples because samples with multiple positive targets were counted separately for each target. 95% CIs were calculated based on a binomial distribution according to Clopper–Pearson.

Species	Any Method (Combined)	Culture	DermaGenius	EUROArray	Culture + DermaGenius	Culture + EUROArray	DermaGenius + EUROArray
Negative/inhibited	n.a.	53	61	50	n.a	n.a.	n.a.
**Dermatophytes**	**65**	**29 (45%)** (95% CI: 32–57)	**58 (89%)** (95% CI: 79–96)	**59 (91%)** (95% CI: 81–97)	**60 (92%)** (95% CI: 83–97)	**61 (94%)** (95% CI: 85–98)	**65 (100%)** (95% CI: 94–100)
*T. interdigitale*/*mentagrophytes*	11	2	11	10	11	10	11
*T. rubrum*/*soudanense*	40	15	35	38	36	38	40
*T. benhamiae*/*erinacei*	2	2	1	2	2	2	2
*T. violaceum*	3	3	3	3	3	3	3
*T. tonsurans*	5	4	5	3	5	4	5
*M. canis*	3	3	3	3	3	3	3
*N. gypsea*	1	0	n.a.	1	0	1	1
**Yeasts**	**16**	**12 (75%)** (95% CI: 48–93)	**3 (19%)** (95% CI: 4–46)	**11 (69%)** (95% CI: 41–89)	**13 (81%)** (95% CI: 54–96)	**16 (100%)** (95% CI: 79–100)	**11 (69%)** (95% CI: 41–89)
*C. albicans*	5	4	3	3	5	5	3
*C. parapsilosis*	9	8	n.a.	6	8	9	6
*C. guilliermondii*	2	0	n.a.	2	0	2	2
**Subtotal (derm. + yeasts)**	**81**	**41 (51%)** (95% CI: 39–62)	**61 (75%)** (95% CI: 64–84)	**70 (86%)** (95% CI: 77–93)	**73 (90%)** (95% CI: 81–96)	**77 (95%)** (95% CI: 88–99)	**76 (94%)** (95% CI: 86–98)
***Fusarium *** **spp.**	**16**	**1 (6%)** (95% CI: 0–30)	**n.a.**	**16 (100%)** (95% CI: 79–100)	**1 (6%)** (95% CI: 0–30)	**16 (100%)** (95% CI: 79–100)	**16 (100%)** (95% CI: 79–100)
*F. solani *(species complex)	12	1	n.a.	12	1	12	12
*F. oxysporum *(species complex)	4	0	n.a.	4	0	4	4
**Subtotal (excl. “other”)**	**97**	**42 (43%)** (95% CI: 33–54)	**61 (63%)** (95% CI: 52–72)	**86 (89%)** (95% CI: 81–94)	**74 (76%)** (95% CI: 67–84)	**93 (96%)** (95% CI: 90–99)	**92 (95%)** (95% CI: 88–98)
Other	25 *	25 *	n.a.	n.a.	25	25	0
**TOTAL detections (excl. negatives)**	**122**	**67 (55%)** (95% CI: 46–64)	**61 (50%)** (95% CI: 41–59)	**86 (70%)** (95% CI: 62–78)	**99 (81%)** (95% CI: 73–88)	**118 (97%)** (95% CI: 92–99)	**92 (75%)** (95% CI: 67–83)

## Data Availability

The data presented in this study are available on request from the corresponding author to ensure that data reuse is scientifically justified and methodologically appropriate.

## Abbreviations

The following abbreviations are used in this manuscript:

PCR	polymerase chain reaction
ITS	internal transcribed spacer
DNA	Deoxyribonucleic Acid
